# Evaluating the feasibility and preliminary efficacy of a Cognitive Occupation-Based programme for people with Multiple Sclerosis (COB-MS): an update to the protocol for a feasibility cluster-randomised controlled trial

**DOI:** 10.1186/s13063-023-07080-y

**Published:** 2023-01-20

**Authors:** Christopher P. Dwyer, Alberto Alvarez-Iglesias, Robert Joyce, Timothy J. Counihan, Dympna Casey, Sinéad M. Hynes

**Affiliations:** 1 Discipline of Occupational Therapy, School of Health Sciences, National University of Ireland Galway, Galway, Ireland; 2 Health Research Board Clinical Research Facility, National University of Ireland Galway, Galway, Ireland; 3 Department of Neurology, University Hospital Galway, Galway, Ireland; 4 Qualitative Research Trials Centre (QUESTS), School of Nursing & Midwifery, National University of Ireland Galway, Galway, Ireland

**Keywords:** Multiple sclerosis, Occupational therapy, Cognitive Occupation-Based programme, Cognitive rehabilitation, Feasibility, Protocol

## Abstract

**Background:**

Cognitive difficulties experienced by people with multiple sclerosis (MS) impact on quality of life and daily functioning, from childcare and work to social and self-care activities. The Cognitive Occupation-Based programme for people with MS (COB-MS) was developed as a holistic, individualised cognitive rehabilitation intervention to address the wide-ranging symptoms and functional difficulties that present in MS, including the ability to maintain employment, social activities, home management and self-care. The aim of the research is to evaluate the feasibility and preliminary efficacy of COB-MS for people with MS.

**Methods:**

Due to the impacts of COVID-19, trial activities that were planned for in-person delivery were completed remotely. One hundred and twenty people with MS will be assigned to participate in either the COB-MS programme or a treatment-as-usual, wait-list control group as part of this single-blind, cluster-randomised controlled feasibility and preliminary efficacy trial of the COB-MS programme. The COB-MS group will participate in an eight-session occupational-based cognitive rehabilitation programme over 9 weeks. The COB-MS intervention was planned for in-person delivery but was delivered online by occupational therapists to small groups of people with MS. The primary outcome measure is the Goal Attainment Scaling at 12 weeks. Participants will be assessed pre-intervention, post-intervention, 12 weeks post-intervention and 6 months post-intervention. Qualitative evaluations of participants’ perspectives will also be examined as part of the feasibility study. Data, due to be collected in-person, was collected online or by post. The original study design, including the statistical analysis plan, remains unchanged despite the shift to a remote trial conduct.

**Discussion:**

Results will provide recommendations for a future definitive trial of COB-MS, with respect to both feasibility and preliminary, clinical efficacy.

**Trial registration:**

ISRCTN ISRCTN11462710. Registered on 9 September 2019 and updated on 23 September 2020 to account for changes outlined here.

## Update

This update describes the amendments made (and approved by the Research Ethics Committee) to a study due to the impact of the COVID-19 pandemic. This update should be read in conjunction with the original published protocol [1].

The trial was halted for approximately 6 months from March 2020 to September 2020. Baseline data collection began in December 2019 and was stopped at the beginning of March 2020. Occupational therapists were trained in-person prior to the trial being halted. The Cognitive Occupation-Based programme for people with Multiple Sclerosis (COB-MS) intervention delivery was due to begin in March 2020. This intervention delivery was halted due to the COVID-19 pandemic. No participants had received the COB-MS intervention.

The research team worked with the Public and Patient Involvement (PPI) Advisory Group and Trial Steering Committee to adapt the trial. The changes that took place are detailed below. The changes involved a full change from in-person to remote data collection and intervention delivery. All changes made to the protocol were approved by Galway University Hospitals on 04.09.2020 and University of Galway Research Ethics Committees 16.09.2020.

## Method

### Design

The original study design—a single-blind, cluster-randomised feasibility trial of the COB-MS programme—remained. Because of the change to online delivery of the COB-MS, the cluster design was no longer essential. The randomisation was, however, completed prior to the COVID-19 pandemic and the feasibility of this design was also being assessed through the trial and therefore no changes were made to the design as a result of the pandemic.

### Participants

#### Setting

The original study settings were participant’s own homes and accessible community venues. These locations were to be used as sites to run the intervention and collect data. Following the arrival of COVID-19 in Ireland and the resultant halting of research activity in March 2020, no further in-person contact took place.

The research remains community-based and will be delivered online due to the impact of COVID-19. Participants will link with the research team and occupational therapists from their own homes, but the COB-MS will be delivered online. The platform used will comply with Health Service Executive policy and standards and The European Data Protection Regulation and be in keeping with NUI Galway data protection policies—e.g. Microsoft Teams, Attend Anywhere and “Zoom for Healthcare”.

Data will be collected in Ireland (online) and remotely via postal response booklets.

#### Recruitment

A number of the occupational therapists originally recruited to deliver the COB-MS intervention were redeployed because of the COVID-19 pandemic. Some occupational therapists, for example, had moved to positions in COVID-19 testing centres, or from community to hospital-based care. Other occupational therapists had significantly increased caseloads because they were covering work and caseloads of those who were redeployed and/or unwell. These issues led to a withdrawal of some occupational therapists from the trial.

This meant that recruitment was re-opened for occupational therapists. Recruitment took place in the same way as originally planned and eligibility criteria remained the same with the exception of the requirement to deliver the COB-MS intervention online rather than in-person.

No further recruitment of participants with MS was necessary. The research team contacted each participant enrolled into the trial to ensure that they wished to continue participation following resumption and that they were able to participate online.

### Measures

No changes were made to the outcomes collected or the associated timings. Baseline data collected in-person from participants prior to March 2020 will not be included in the final analysis given the timeframe that elapsed before intervention delivery (or control equivalent). All participants will be re-assessed remotely, as described below.

All data will be collected remotely. Self-report questionnaires will be completed by participants either on paper (and posted back using a self-addressed and stamped envelope) or online through Microsoft Forms. Other outcomes will be completed online or over the phone with participants.

Participants will be posted the response sheet booklets in advance of data collection sessions and complete the outcomes via video call in conjunction with the research assistant. The response sheet will be in a separate envelope not opened until instructed by the research assistant. Barcellos et al. [2] reported remote testing of SDMT and CVLT II to be equivalent and reliable for people with MS. We further tested this with other cognitive measures [3] and found this method of data collection to be equivalent.

### Interventions

#### COB-MS (experimental) condition

The COB-MS was initially planned to take place in a community setting but this has moved online due to COVID-19. Both individual and group sessions will take place online. Both the participant and the occupational therapist will ensure that they have privacy for the session. This is especially important for the participants during the group sessions. Each occupational therapist will run the COB-MS with 5–6 participants—this group size has reduced to allow for online delivery, as suggested by the COB-MS PPI group.

Any occupational therapist who took part in an in-person COB-MS training day will have a refresher training online. All newly recruited occupational therapists will be trained online. All of the occupational therapists delivering the COB-MS will have access to training videos and material, as well as resources that can be used in the delivery of the online COB-MS sessions.

Participants with MS in the experimental group will receive a physical copy of the COB-MS handbook in the post.

#### Control condition

No change was made to the control condition. All participants, regardless of their allocation, were contacted to reconfirm consent once the changes to delivery were explained. The control condition will be offered the intervention online following the completion of data collection.

### Statistical analysis

The statistical analysis plan remains unchanged from the initial protocol.

## Current status of the study

This adapted protocol was approved and implemented in September 2020. Data collection was completed in November 2021. Trial result reporting is underway.

## Data Availability

Data sharing is not applicable to this article as no datasets were generated or analysed for this protocol. Data and materials will be made available from the trial once completed and reported on.

## Abbreviations

COB-MS: A Cognitive Occupation-Based programme for people with Multiple Sclerosis
MS: Multiple sclerosis
PPI: Public an Patient Involvement
